# Genome-Wide Identification of Xyloglucan Endotransglucosylase/Hydrolase Multigene Family in Chinese Jujube (*Ziziphus jujuba*) and Their Expression Patterns Under Different Environmental Stresses

**DOI:** 10.3390/plants13243503

**Published:** 2024-12-15

**Authors:** Mohamed Refaiy, Muhammad Tahir, Lijun Jiao, Xiuli Zhang, Huicheng Zhang, Yuhan Chen, Yaru Xu, Shuang Song, Xiaoming Pang

**Affiliations:** 1 State Key Laboratory of Tree Genetics and Breeding, National Engineering Research Center of Tree Breeding and Ecological Restoration, Key Laboratory of Genetics and Breeding in Forest Trees and Ornamental Plants, Ministry of Education, College of Biological Sciences and Biotechnology, Beijing Forestry University, Beijing 100083, China; m_refaiy@bjfu.edu.cn (M.R.); malikuaf2011@gmail.com (M.T.); chenyuhan2023@bjfu.edu.cn (Y.C.); 17862824095@163.com (Y.X.); shuangsong17@163.com (S.S.); 2 Shuangjing Forest Farm, Aohan Banner, Chifeng 028000, China; jiaolijun12345@163.com; 3 Xinhui Forest Farm, Aohan Banner, Chifeng 028000, China; nmcxx2008@163.com; 4 Agricultural and Animal Husbandry Technology Promotion Service Center, Aohan Banner, Chifeng 028000, China; cfcxx2006@163.com

**Keywords:** xyloglucan endotransglucosylase/hydrolase (XTH), salt stress, drought stress, cold stress, Chinese jujube

## Abstract

The Xyloglucan endotransglucosylase/hydrolase (XTH) family, a group of cell wall-modifying enzymes, plays crucial roles in plant growth, development, and stress adaptation. The quality and yield of Chinese jujube (*Ziziphus jujuba*) fruit are significantly impacted by environmental stresses, including excessive salinity, drought, freezing, and disease. However, there has been no report of the XTH encoding genes present in the Chinese jujube genome and their response transcription level under various stresses. This study provides an in-depth analysis of *ZjXTH* genes in the genome of Chinese jujube and elucidates their structural motifs, regulatory networks, and expression patterns under various stresses. A total of 29 *ZjXTH* genes were identified from the *Ziziphus jujuba* genome. Phylogenetic analysis classifies *ZjXTH* genes into four distinct groups, while conserved motifs and domain analyses reveal coordinated xyloglucan modifications, highlighting key shared motifs and domains. Interaction network predictions suggest that *ZjXTHs* may interact with proteins such as Expansin-B1 (EXPB1) and Pectin Methylesterase 22 (PME22). Additionally, cis-regulatory element analysis enhances our understanding of Chinese jujube plant’s defensive systems, where TCA- and TGACG-motifs process environmental cues and orchestrate stress responses. Expression profiling revealed that *ZjXTH1* and *ZjXTH5* were significantly upregulated under salt, drought, freezing, and phytoplasma infection, indicating their involvement in biotic and abiotic stress responses. Collectively, these findings deepen our understanding of the functional roles of Chinese jujube *XTHs*, emphasizing their regulatory function in adaptive responses in Chinese jujube plants.

## 1. Introduction

The plant cell wall, an essential structure external to the cell membrane, is a dynamic and adaptable component that can be rapidly remodeled in response to both developmental and environmental signals. It is predominantly composed of polysaccharides, including cellulose, hemicellulose, pectin, and glycoproteins. The cell wall serves multiple critical functions, such as determining plant morphology, providing structural integrity, offering mechanical support, and defending against biotic and abiotic stresses [1,2,3]. In recent years, growing evidence has highlighted that cell wall loosening and reorganization are pivotal events in processes such as cell proliferation, volumetric expansion, morphological changes, and stress adaptation [4]. Of particular significance are cell wall-modifying proteins, which play a crucial role in structural remodeling, enabling plants to respond and adapt to fluctuating environmental conditions, including biotic and abiotic stress [4,5,6]. The Xyloglucan endotransglucosylase/hydrolase (*XTH*) gene family plays a pivotal role in the plasticity and remodeling of the plant cell wall by mediating the cleavage and rejoining of xyloglucan polymers. Along with members of the glycoside hydrolase family 16 (GH16), XTH enzymes are thought to modulate cell wall mechanics and expansion through these catalytic activities [7,8,9]. The XTH family exhibits two primary enzymatic functions: xyloglucan endotransglucosylase (XET) activity, responsible for xyloglucan polymer rearrangement, and xyloglucan endohydrolase (XEH) activity, which hydrolyzes xyloglucan glycosidic bonds to facilitate cell wall expansion and morphogenesis [7]. Structurally, XTH proteins are categorized into four distinct groups: I/II, III A, III B, and an ancestral group. Enzymes within Group I/II primarily demonstrate glycosyltransferase activity, while those in Group III are predominantly associated with hydrolase activity [10,11]. GH16 family members characteristically contain the conserved Glyco_hydro_16 motif and an N-glycosylation site, which are essential for their enzymatic function [12]. With advancements in plant genome sequencing, the identification and functional regulation of XTH family members have been extensively studied in various plant species, including *Arabidopsis thaliana* [10], *Oryza sativa* [13], *Solanum lycopersicum* [14], *Glycine max* [15], *Hordeum vulgare*, and two Brassica species (*Brassica rapa* and *Brassica oleracea*) [16], as well as *Camellia sinensis* [17]. Additionally, various plant hormones modulate the expression of Xyloglucan Endotransglucosylase/Hydrolase (*XTH*) genes. Auxins, ethylene, gibberellins, abscisic acid, brassinosteroids, and cytokinins play a complex role in regulating XTH activity, thereby impacting cell wall architecture, growth processes, and defense mechanisms.

Extensive research has established the critical roles of *XTHs* (xyloglucan endo-transglucosylase/hydrolases), thereby impacting cell wall architecture, growth, and defense mechanisms. For example, the overexpression of *CaXTH3* in both tomato and Arabidopsis has been shown to enhance salt and drought tolerance by regulating stomatal closure in transgenic plants [18,19]. The constitutive expression of *PeXTH* in tobacco conferred increased resistance to salt and cadmium [12]. In Arabidopsis, loss-of-function mutations in *XTH15*, *XTH17*, and *XTH31* resulted in heightened aluminum tolerance compared to wild-type controls [20]. Additionally, *AtXTH30* has been identified as a negative regulator of salt tolerance, influencing xyloglucan side chain modification, the concentration of xyloglucan-derived oligosaccharides, cellulose biosynthesis, and the stability of cortical microtubules [21]. Furthermore, *XTH19* and *XTH23* are implicated in lateral root development through the BES1-dependent pathway, facilitating the adaptation of lateral roots to salt stress [22]. Notably, the *XTH19* mutant exhibited reduced freezing tolerance following cold and sub-zero acclimation, attributed to alterations in cell wall composition and structure [23]. In *Brachypodium distachyon*, a monocot model species, increased expression of *XTH* genes and those involved in Xylan biosynthesis has been closely linked to enhanced drought tolerance [5].

Chinese jujube (*Ziziphus jujuba* Mill.), native to China, is a vital fruit crop for commerce, the environment, and social value, especially in central and western China. With a global growing area exceeding 1.5 million hectares, China remains the sole supplier, supporting over 1000 recognized cultivars [24]. The top cultivars are usually graft-propagated, and traits like drought and saline-alkali resistance are largely dependent on the rootstock. Cold tolerance and resistance to Jujube witches’ broom (JWB) disease are essential for selecting robust cultivars. Under low saline-alkali stress (0–90 mM), jujube fruit shows increased sucrose, glucose, and fructose levels, along with enhanced activities of sucrose phosphate synthase. However, high saline-alkali stress (120–150 mM) inhibits sugar accumulation [25]. Drought stress, seen at soil moisture levels of 30–50% field capacity, significantly reduces fruit redness and lowers anthocyanin and carotenoid levels [26]. Additionally, severe freezing stress enhances the galactose metabolism pathway [27]. Jujube witches’ broom (JWB) disease, caused by phytoplasma infection, leads to substantial crop losses in the woody perennial fruit tree *Z*. *jujuba* [18]. The presence of JWB-associated phytoplasmas decreases auxin accumulation and downregulates the expression of *ZjARF4* in flower tissues, leading to phyllody symptoms. *ZjTCP6* and *ZjTCP16* are likely key regulatory factors involved in the jujube plant’s response to JWB [28]. Additionally, *PHYL1* promotes the proteasome-mediated degradation of crucial floral morphogenetic regulators in both jujube and Arabidopsis [29]. Moreover, *CsXTH04* was identified as a potential candidate gene, upregulated in citrus varieties resistant to bacterial canker (*Xanthomonas citri* subsp. *citri*) and induced by exogenous treatments with salicylic acid and methyl jasmonate [30].

Genome-wide identification is critical to contemporary crop breeding strategies, as it helps identify candidate genes associated with traits critical to agriculture productivity and nutrition. Similarly, RNA-Seq analysis is an effective approach for determining gene involvement and expression patterns across various biological processes, organs, and cell types. This method has proven valuable for studying genes involved in defense mechanisms and their specialized functions. The present research employs in silico analysis to investigate the *ZjXTH* gene family in Chinese jujube and to understand how these genes contribute to the plant’s defense against biotic and abiotic stress factors. Examining *ZjXTH* expression patterns, transcriptional networks, and stress-responsive elements advances our understanding of plant adaptability and provides a foundation for genetic interventions aimed at enhancing stress tolerance in jujube cultivation.

## 2. Results

### 2.1. Identification and Sequence Analysis of XTH Genes

We successfully identified the *XTH* family genes in the *Z. jujuba* ‘Dongzao’ genome available in the NCBI database under accession ASM3175591v1. Subsequent validation through BLAST-P, using 33 *AtXTH* proteins as references, revealed a total of 29 potential *ZjXTH* genes. These genes are predicted to encode proteins containing both the Glyco_hydro_16 domain (PF00722) and the XET_C domain (PF06955). The genes were designated *ZjXTH1* through *ZjXTH29* based on their chromosomal locations (Table 1). The Expasy online platform was employed to analyze the properties of *ZjXTH* proteins, including their amino acid length (AA), aliphatic index (AI), instability index (II), molecular weight (MW), and theoretical isoelectric point (pI). Signal peptides (SPs) were predicted using the SignalP 6.0 Service. The results showed that the amino acid lengths ranged from 271 to 352, while the gene sequences varied from 815 to 1045 base pairs. The molecular weights of the proteins ranged from 30 to 40 kDa, corresponding to their respective lengths. The predicted isoelectric points (pI) ranged from 5.24 to 9.38, which may be attributed to differences in the polarity of the amino acids. Furthermore, 25 of the *ZjXTH* proteins were predicted to contain signal peptides, while 4 lacked any signal peptide. Notably, an analysis of the grand average of hydropathy (GRAVY) values for these proteins revealed negative scores, ranging from −0.137 to −0.713, indicating a predominantly hydrophilic nature. Additional metrics, such as the instability index (II) and aliphatic index (AI), are also presented in Table 1.

### 2.2. Phylogenetic Classification of ZjXTH, Their Motif, and Gene Structure Analysis

**Figure 1:**
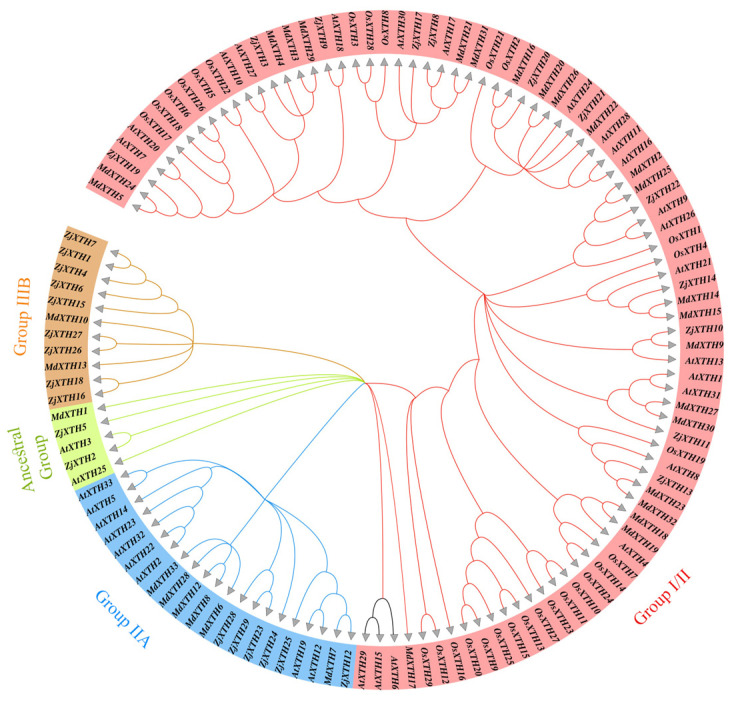
Phylogenetic analysis of XTH proteins among 29 *ZjXTHs* from *Ziziphus jujuba*, 33 *AtXTHs* from *Arabidopsis thaliana*, 29 *OsXTHs* from *Oryza sativa*, and 15 *MdXTHs* from *Malus domestica*. Whole protein sequences of the *XTHs* gene family were used for alignment using MEGA X software. The phylogenetic tree was constructed ussssing the IQ-TREE 2 web tool using maximum likelihood with 1000 bootstrap replicates. Different-colored branches correspond to distinct XTH subfamilies, and the XTH IDs of arabidopsis, apple, and rice were assigned based on previous studies.

A maximum likelihood analysis was performed to investigate the interspecific and intraspecific phylogenetic relationships among *ZjXTH* protein sequences. A total of 106 XTH proteins were compiled from four distinct species. To assess the evolutionary relationships, a phylogenetic tree was constructed using 29 *ZjXTH* proteins from *Ziziphus jujuba*, 33 *AtXTH* proteins from *Arabidopsis thaliana*, 29 *OsXTH* proteins from *Oryza sativa*, and 15 *MdXTH* proteins from *Malus domestica*. Phylogenetic analysis revealed that the 29 *ZjXTH* proteins were grouped into four distinct clades. Twelve *ZjXTH* proteins were classified under Group I/II, nine were grouped in the IIIB subgroup, six in Group IIA, and two *ZjXTH* proteins were placed in the ancestral group (Figure 1).

**Figure 2:**
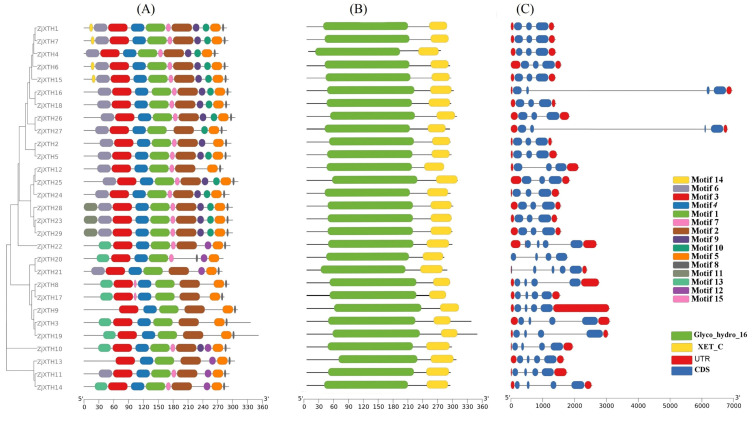
Comparative analysis of the phylogenetics, exon–intron structures, and conserved motifs of the XTH family in Chinese jujube (*ZjXTHs*). (**A**) Motif composition models of 29 XTH proteins, with different motifs color-coded according to the legend. (**B**) Two conserved domains were identified and are represented in green and yellow. (**C**) The gene structures of *ZjXTH* were analyzed and visualized, including introns (black lines), exons (coding sequences, blue rectangles), and untranslated regions (UTRs, red rectangles).

To compare the *ZjXTHs* at both the nucleic acid and protein levels, we conducted a comprehensive analysis of the conserved motifs, gene structure, domains, and phylogenetic relationships among the 29 *ZjXTH* proteins (Figure 2). Using the MEME search tool, we identified fifteen conserved motifs labeled as motifs 1–15. The results showed varying compositions and distributions of these motifs among the *ZjXTH* proteins. Motifs 1 through 6 were identified in all *ZjXTH* proteins in the same arrangement. In contrast, motif 11 was found only in *ZjXTH23*, *ZjXTH28*, and *ZjXTH29*, while motif 14 appeared exclusively in *ZjXTH1*, *ZjXTH6*, *ZjXTH7*, and *ZjXTH15* (Figure 2A). We also used the NCBI CD-blast tool to analyze the conserved domains of the *ZjXTH* proteins and found that the XTH domain was present in all 29 proteins. Interestingly, two other domains, Glyco_hydro_16 and the XET_C superfamily, were consistently present across all gene families (Figure 2B). Our investigation into the genetic architecture of the *XTH* genes revealed differences in the number of exons and introns they contain. In general, genes grouped together tend to share similar structures. For instance, all members of Group 1 (*ZjXTH1*, *ZjXTH2*, *ZjXTH3*, *ZjXTH4*, *ZjXTH5*, *ZjXTH6*, *ZjXTH9*, *ZjXTH12*, *ZjXTH15*, *ZjXTH17*, *ZjXTH18*, *ZjXTH21*, *ZjXTH23*, *ZjXTH24*, *ZjXTH25*, *ZjXTH26*, *ZjXTH28*, and *ZjXTH29*) contained three exons and two introns in their coding regions. In contrast, members of Group 2 (*ZjXTH16*, *ZjXTH27*, *ZjXTH20*, *ZjXTH22*, *ZjXTH7*, *ZjXTH8*, *ZjXTH10*, *ZjXTH11*, *ZjXTH13*, *ZjXTH14*, and *ZjXTH19*) possessed four exons and three introns. Additionally, it is noteworthy that within this group, *ZjXTH16* and *ZjXTH27* stand out, as they possess the longest introns among the identified genes (Figure 2C).

### 2.3. Chromosomal Localization, Synteny Analysis, and PPI of ZjXTH Genes

**Figure 3:**
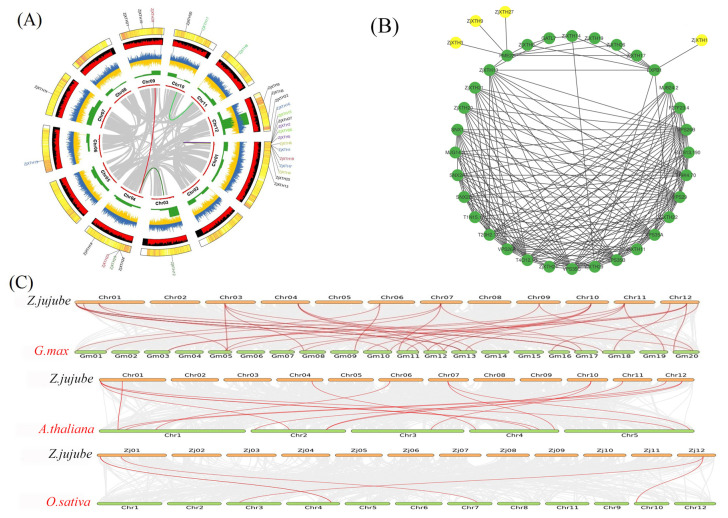
Chromosomal localization and synteny analysis of *ZjXTH* proteins in the Chinese jujube genome. Genes IDs in black indicate an absence of collinearity, genes and lines colored in green indicate dispersed duplication, red indicates whole genome duplication, and blue-colored lines indicate transposed duplicated pairs (**A**). Protein–protein interaction analyses were performed using the String web tool and visualized using Cytoscape software v3.10.3. The network consists of various proteins represented as nodes, with interactions depicted by edges. Proteins highlighted in yellow form key hubs with multiple interactions, suggesting their significant role in the network. Green nodes represent additional interacting proteins (**B**). Syntenic relationships of *ZjXTH* genes between *Arabidopsis thaliana*, *Glycine max*, and *Oryza sativa*. The brown lines in the background represent the collinear blocks within *Ziziphus jujuba* and other plant genomes, while the red lines highlight the syntenic *ZjXTH* gene pairs (**C**).

To understand the chromosomal distribution of the Chinese jujube *XTH* genes, we physically mapped the locations of the 29 *ZjXTH* genes on the Chinese jujube genome. This investigation revealed a non-uniform distribution pattern across the chromosomes. Chromosome 01 harbored the highest number of *ZjXTH* genes [13], followed by Chromosomes 03, 06, 07, and 11, each containing one *ZjXTH* gene. *ZjXTH* genes were also located on Chromosomes 09 and 12, with three genes on each. Chromosome 10 contained two *ZjXTH* genes, while four *ZjXTH* genes were found on Chromosome 04 (Figure 3A).

Protein–protein interaction (PPI) analyses have revealed that several XTH proteins, including *ZjXTH1*, *ZjXTH3*, *ZjXTH8*, *ZjXTH9*, *ZjXTH10*, *ZjXTH13*, *ZjXTH14*, *ZjXTH27*, and *ZjXTH29*, are positively involved in the metabolism of polysaccharides, particularly glucuronarabinoxylan. Notably, *ZjXTH1* interacts with Expansin-B1 (EXPB1), a protein crucial for cell wall loosening, which facilitates processes requiring cell wall modification. This interaction also contributes to the formation of root hairs, which are vital for efficient water and nutrient uptake. Additionally, *ZjXTH3*, *ZjXTH9*, and *ZjXTH27* interact with Pectin Methylesterase 22 (PME22), which plays a pivotal role in the demethylesterification of pectin. This modification of cell wall properties is integral to the plant’s response to various environmental stresses, including salt stress, drought, and pathogen attack. These findings underscore the diverse and essential functions of XTH proteins in *Ziziphus jujuba* and highlight their potential roles in the physiological adaptations of Chinese jujube plants (Figure 3B).

In this study, we utilized the MCScanX method to delve into the phenomenon of gene duplication within the *ZjXTH* gene family. In order to conduct a thorough investigation of the potential evolutionary pathways associated with the *ZjXTH* gene family, we have constructed three comparative syntenic maps. These maps include *Ziziphus jujuba*, a dicotyledonous plant, alongside monocotyledonous species such as *Arabidopsis thaliana*, *Glycine max*, and *Oryza sativa* (Figure 3C). As a result, a comprehensive set of orthologous genes was identified, consisting of thirty-four pairs shared between *Ziziphus jujuba* and *Glycine max*, seventeen collinear gene pairs belonging to the *ZjXTH* gene family between *Ziziphus jujuba* and *Arabidopsis thaliana*, and four orthologous genes shared between *Ziziphus jujuba* and *Oryza sativa*. The higher frequency of orthologous events detected in *GmXTHs-ZjXTHs*, relative to other orthologous events, indicates a shorter evolutionary gap between *Ziziphus jujuba* and *Glycine max*. The evolutionary significance of *XTH* genes may have been influenced by the existence of a minimum of four pairs of orthologous genes in select *OsXTH* genes. The results of this investigation suggest that the *ZjXTH* genes identified in *Ziziphus jujuba* could have derived from orthologous genes present in other plant species (Figure 3C).

### 2.4. Cis-Regulatory Elements in the Promoter Region of ZjXTH

**Figure 4:**
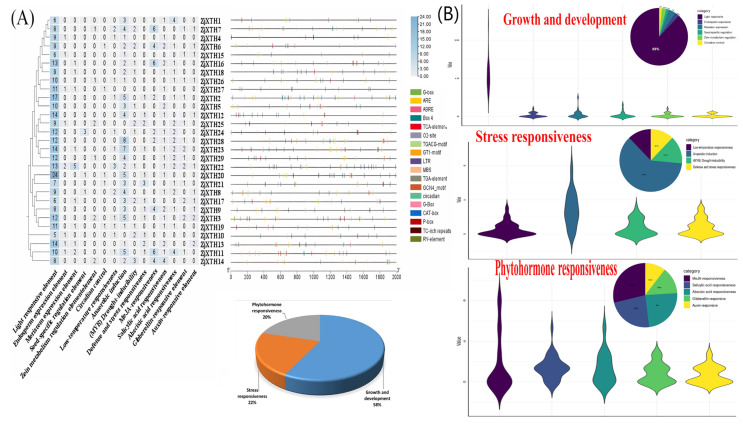
Analysis of cis-regulatory elements (CREs) in the putative promoter region of *ZjXTH* genes using the PlantCARE database. (**A**) The number of predicted CREs located in the 2k bp upstream of the *ZjXTH* genes and the distribution of the three categories of CREs among the members of the *ZjXTH* gene family. (**B**) Venn diagram plot and pie chart showing the distribution of different functional categories of CREs identified in the *ZjXTH* promoter region.

The upstream region, consisting of 2 kb nucleotides from the start codon, was analyzed to investigate the promoter cis-regulatory elements (CREs). The data were submitted for analysis through the PlantCARE database. The results revealed a total of 607 CREs distributed across all *ZjXTH* genes, with *ZjXTH20* containing the highest number at 33, and *ZjXTH10* possessing the fewest at 10 CREs. These elements were systematically classified into three main categories: regulatory elements involved in growth and development, stress-responsive elements, and phytohormone-responsive elements, as visually illustrated for clearer understanding (Figure 4).

(i) The regulatory elements associated with growth and development represented the largest proportion of CREs within the promoter regions of the *ZjXTH* genes, comprising 350 elements and accounting for 58% of the total CREs. All *ZjXTH* genes contained elements from this category, predominantly those linked to light responsiveness. Specifically, light-responsive CREs, such as G-box, Box II, Box 4, TCT-motif, and I-box, were highly prevalent, making up 308 elements, which constituted 88% of the total elements in this category. Additional regulatory elements included meristem expression elements (CAT-box) with 14 elements (4%), endosperm expression elements (GCN4_motif), and zein metabolism regulatory elements (O2-site), each with nine elements (2.5%). Furthermore, cis-elements associated with circadian control and seed-specific regulation were present in five elements (1.5%) each across the promoters of all *ZjXTH* genes.
(ii) The second category comprises stress-responsive CREs, with a total of 132 elements (22%) identified in the promoter regions of the *ZjXTH* genes. At least one CRE from this category was found in every *ZjXTH* gene. Among these, anaerobic induction elements (ARE) were the most abundant, with 81 elements (61.4%). This was followed by drought-inducibility elements (MBS), comprising 19 elements (14.4%), while defense and stress-responsive elements (TC-rich repeats) and low-temperature responsive elements (LTR) were equally represented, each contributing 16 elements (12.1%).
(iii) The third category of CREs identified in the promoter regions of *ZjXTH* genes is associated with phytohormone responsiveness, comprising a total of 125 predicted CREs (20%). The most abundant of these elements is the MeJA-responsive motif (TGACG-motif), which accounts for 36 CREs (28.8%). This is followed by abscisic acid-responsive elements (ABRE) with 30 CREs (24%) and salicylic acid-responsive elements (TCA-element) with 29 CREs (23.2%). Gibberellin-responsive elements contributed 17 CREs (13.6%), while auxin-responsive elements (TGA-element) represented the smallest proportion, with 13 CREs (10.4%).

### 2.5. Functional GO Annotation and Ka/KS Analysis of ZjXTHs

**Figure 5:**
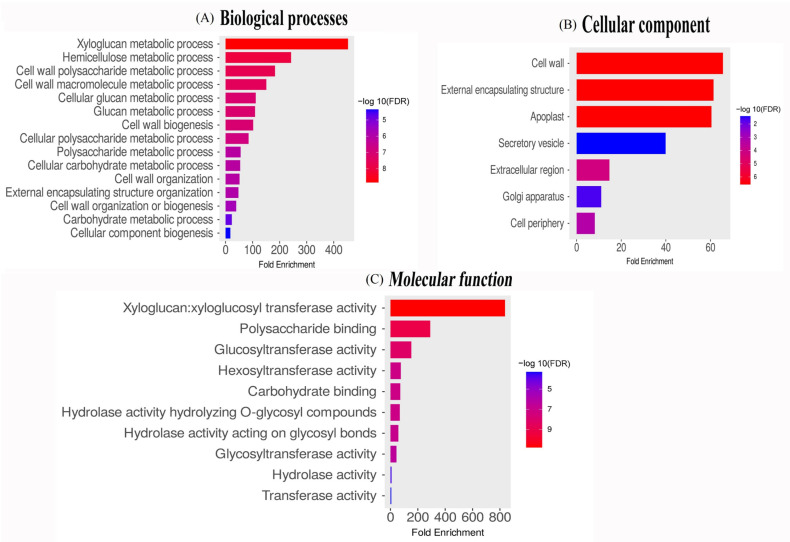
Gene ontology (GO) analysis was conducted on the *ZjXTH* gene family to assess its functional distribution across the genome. GO annotations were assigned to the *ZjXTH* gene sequences, categorizing them into three primary domains: (**A**) biological process, (**B**) cellular component, and (**C**) molecular function. The resulting bar graph illustrates the proportional distribution of *ZjXTH* genes across these categories, providing insights into their potential roles in various biological pathways and cellular functions.

The functional annotation of *ZjXTH* genes identified 14 distinct gene ontology (GO) terms, classified into three major categories: molecular function, biological process, and cellular component. The molecular function of *ZjXTH* genes was predominantly linked to xyloglucosyl transferase activity (GO:00167762), aligning with the established role of *ZjXTH* genes in xyloglucan modification. In terms of cellular localization, *ZjXTH* genes were mainly associated with the cell wall (GO:005618), external encapsulating structure (GO:0030312), and apoplast (GO:0048046), highlighting their critical role in the structural development of Chinese jujube (*Ziziphus jujuba*) plants. Under the biological process category, *ZjXTH* genes were implicated in regulating metabolic pathways, including xyloglucan (GO:0010411) and hemicellulose metabolism (GO:0010410). Additionally, several *ZjXTH* genes were linked to the positive regulation of cellular processes and developmental pathways. Collectively, these findings underscore the significant contributions of *ZjXTH* genes to the growth and development of Chinese jujube plants (Figure 5).

To gain a comprehensive insight into the evolutionary dynamics and selective pressures influencing the *ZjXTH* gene family, we calculated the nonsynonymous-to-synonymous substitution rate ratio (Ka/Ks) for all 29 *ZjXTH* genes within the Chinese jujube (*Ziziphus jujuba*) genome. These results are systematically detailed in Table 2. In the context of evolutionary selection, a Ka/Ks value of less than 1 indicates purifying selection, while values exceeding 1 suggest positive or diversifying selection. Two distinct gene groups emerged from our analysis: the first group, including gene pairs such as *ZjXTH2-ZjXTH5* and *ZjXTH4-ZjXTH6*, displayed Ka/Ks values ranging from 0.11 to 0.9, indicating strong purifying selection. The second group, represented by gene pairs *ZjXTH15-ZjXTH26* and *ZjXTH17-ZjXTH8*, exhibited Ka/Ks ratios between 1.18 and 1.54, suggesting a more pronounced role of positive selection. Overall, these findings imply that purifying selection has predominantly shaped the evolutionary trajectory of *ZjXTH* genes, preserving their conserved structural features and highlighting their critical functional roles within the genome.

### 2.6. Expression Patterns of ZjXTHs on Subcellular Levels and Different Tissues

**Figure 6:**
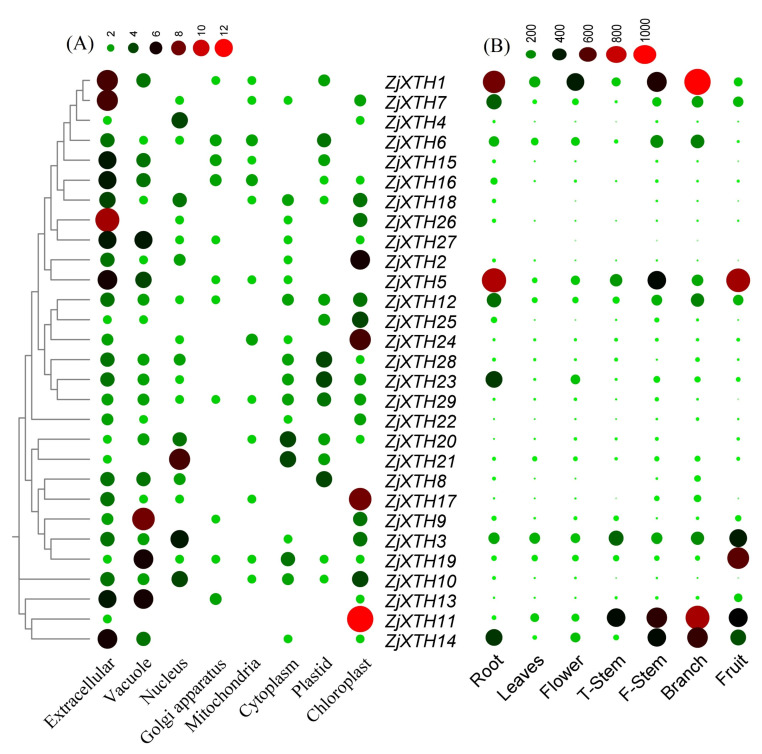
Heatmaps were generated to examine the expression patterns of *ZjXTHs* under various cellular compartments, developmental stages, and stress conditions. The heatmaps were constructed and visualized using TBTools software v2.102. (**A**) The sub-cellular localization of *ZjXTH* proteins was predicted using the WoLF PSORT web tool. (**B**) The tissue-specific expression profiles of *ZjXTH* at different developmental stages of the Chinese jujube plant were analyzed using publicly available transcriptome data and displayed in a heatmap. The normalized fragments per kilobase of transcript per million fragments (FPKM) values. A deeper red indicates higher expression levels, while a deeper green represents lower expression levels.

To determine the subcellular localization of *ZjXTH* proteins, we employed the WoLF PSORT online database. The analysis revealed that the majority of *ZjXTH* proteins were predominantly localized to the extracellular space (Figure 6A), indicating their potential role as catalysts in decomposition processes. Specifically, *ZjXTH1*, *ZjXTH5*, *ZjXTH7*, *ZjXTH14*, and *ZjXTH26* were predicted to be exclusively localized in the extracellular space. Additionally, certain *ZjXTH* proteins were predicted to localize to other subcellular compartments. For instance, *ZjXTH11* and *ZjXTH17* were significantly localized in the chloroplast, while *ZjXTH20* and *ZjXTH21* were predicted to localize in both the cytoplasm and nucleus. *ZjXTH9* was primarily found in the vacuole, and *ZjXTH8*, *ZjXTH23*, and *ZjXTH28* showed traces of localization within plastids.

We investigated the expression profile of *ZjXTHs* in various tissues using publicly available transcriptome data by RNA-Seq. The tissues analyzed included the roots, leaves, flowers, T-stems, F-stems, branches, and fruit as shown in (Figure 6B); we observed significant differences in the expression patterns of *ZjXTH* transcripts. Most *ZjXTH* genes, including *ZjXTH1*, *ZjXTH5*, and *ZjXTH14*, exhibited high expression levels in vegetative tissues such as roots, flowers, F-stems, and branches. In contrast, *ZjXTH5* demonstrated notably high expression in fruit, with the highest levels observed in the T-stem, indicating its potential importance in tissue development. The expression of *ZjXTH1* was absent across all tissues analyzed. Furthermore, *ZjXTH2*, *ZjXTH4*, *ZjXTH15*, *ZjXTH18*, *ZjXTH26*, *and ZjXTH27* showed minimal expression, suggesting a lesser role in organ development. While most *ZjXTH* genes exhibited consistent tissue-specific expression patterns, *ZjXTH27* was uniquely downregulated across all tissues. Notably, *ZjXTH3*, *ZjXTH7*, *ZjXTH11*, and *ZjXTH12* were predominantly expressed in roots, leaves, flowers, T-stems, F-stems, branches, and fruit. Among these, *ZjXTH19* displayed the highest expression in fruit. These results highlight a distinct, tissue-specific expression profile for *ZjXTH* genes, suggesting their specialized roles in various tissues and developmental processes.

### 2.7. Expression Profiles of ZjXTH Under Salt Stress

**Figure 7:**
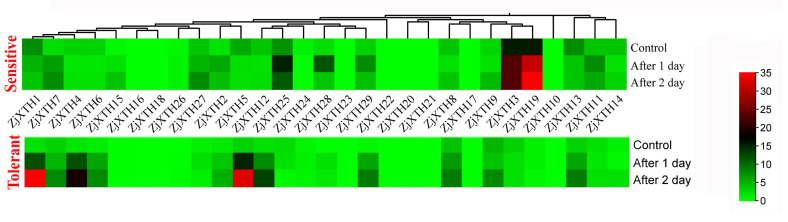
Expression patterns of *ZjXTHs* of 29 differentially expressed genes in *Z. jujuba*. var. spinosa diploid and tetraploid seedlings, representing sensitive and tolerant types, respectively, were used in a salinity treatment, gradually applied at 50, 100, and 150 mM NaCl. A deeper red indicates higher expression levels, while a deeper green represents lower expression levels.

A total of 29 *ZjXTH* genes were identified, and their expression patterns under salt stress were thoroughly analyzed. The findings revealed significant temporal differences in the expression of these genes in response to salt stress. *ZjXTH1* and *ZjXTH5* were notably upregulated after one and two days of salt treatment in the salt-tolerant tetraploid sour, displaying the most rapid and robust response. These genes were the most highly expressed, suggesting their critical roles in the salt stress response. In contrast, *ZjXTH6*, *ZjXTH7*, *ZjXTH12*, and *ZjXTH13* were downregulated under the same conditions. Some genes exhibited negatively regulated expression patterns. For instance, the expression of *ZjXTH3* and *ZjXTH19* remained relatively unchanged in the tolerant cultivar during both control and salt stress treatments, but significantly increased in the sensitive cultivar. Additionally, *ZjXTH10*, *ZjXTH16*, *ZjXTH18*, *ZjXTH20*, and *ZjXTH21* showed no detectable expression in either cultivar, indicating that they may not play a pivotal role in the salt stress response (Figure 7).

### 2.8. Expression Profiles of ZjXTH Under Drought Stress

**Figure 8:**
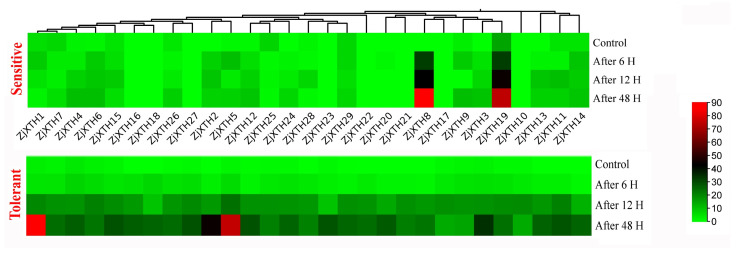
Expression patterns of 29 differentially expressed *ZjXTH* genes were analyzed in diploid and tetraploid *Z. jujuba*. var. spinosa seedlings, representing sensitive and tolerant types, respectively, under PEG6000 concentrations of 5%, 10%, 15%, and 20% applied over 1-day intervals. The heatmaps represent the average FPKM values of the genes. A deeper red indicates higher expression levels, while a deeper green represents lower expression levels.

The results revealed substantial temporal variations in the expression of *ZjXTH* genes in response to drought stress. *ZjXTH1* and *ZjXTH5* were highly expressed at 6, 12, and 48 h following drought treatment in the drought-tolerant jujube cultivar, demonstrating the most rapid and pronounced response. These genes exhibited the highest expression levels, indicating their pivotal roles in the drought stress response. Conversely, *ZjXTH2*, *ZjXTH4*, *ZjXTH6*, *ZjXTH12*, and *ZjXTH15* were downregulated under the same conditions. They showed relatively stable expression during drought stress treatments but were significantly upregulated in the tolerant cultivar. Furthermore, *ZjXTH8* and *ZjXTH19* exhibited negatively regulated expression patterns (Figure 8).

### 2.9. Expression Profiles of ZjXTH Under Freezing Stress

**Figure 9:**
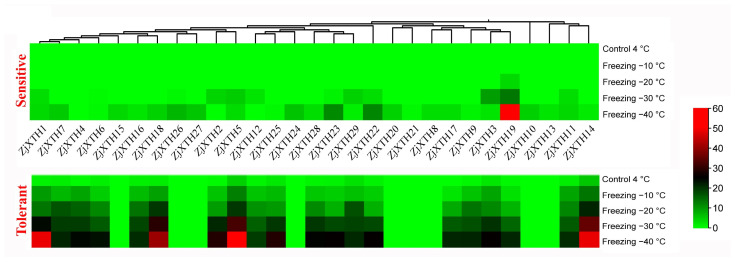
Expression patterns of 29 differentially expressed *ZjXTH* genes were analyzed in the cold-sensitive cultivar ‘Dongzao’ and the cold-tolerant cultivar ‘Jinsixiaozao’. A deeper red indicates higher expression levels, while a deeper green represents lower expression levels.

We analyzed the gene expression patterns of *ZjXTHs* under cold stress, as presented in Figure 9. The analysis revealed six distinct stages of differential gene expression. In the first stage, *ZjXTH1*, *ZjXTH7*, *ZjXTH4*, and *ZjXTH6* were moderately expressed under freezing conditions, with *ZjXTH1* exhibiting a notably higher expression level. In the second stage, *ZjXTH6* and *ZjXTH18* were moderately expressed, while *ZjXTH8* showed a significant increase in expression, particularly in cold-tolerant cultivars. During the third stage, *ZjXTH2*, *ZjXTH12*, and *ZjXTH25* were downregulated in cold-sensitive cultivars but upregulated in cold-tolerant cultivars. Notably, *ZjXTH5* was highly expressed. In stages four and five, *ZjXTH28*, *ZjXTH23*, *ZjXTH29*, *ZjXTH22*, *ZjXTH17*, and *ZjXTH3* exhibited downregulation in tolerant cultivars, while *ZjXTH19* was negatively regulated. Additionally, in the six *ZjXTH* stages, *ZjXTH11* and *ZjXTH14* were downregulated, except for *ZjXTH14*, which was highly expressed in cold-tolerant cultivars. Furthermore, *ZjXTH21* showed no expression in either cultivar, suggesting that this gene may not play a critical role in the cold stress response.

### 2.10. Expression Profiles of ZjXTH Response to Phytoplasma Infection

**Figure 10:**
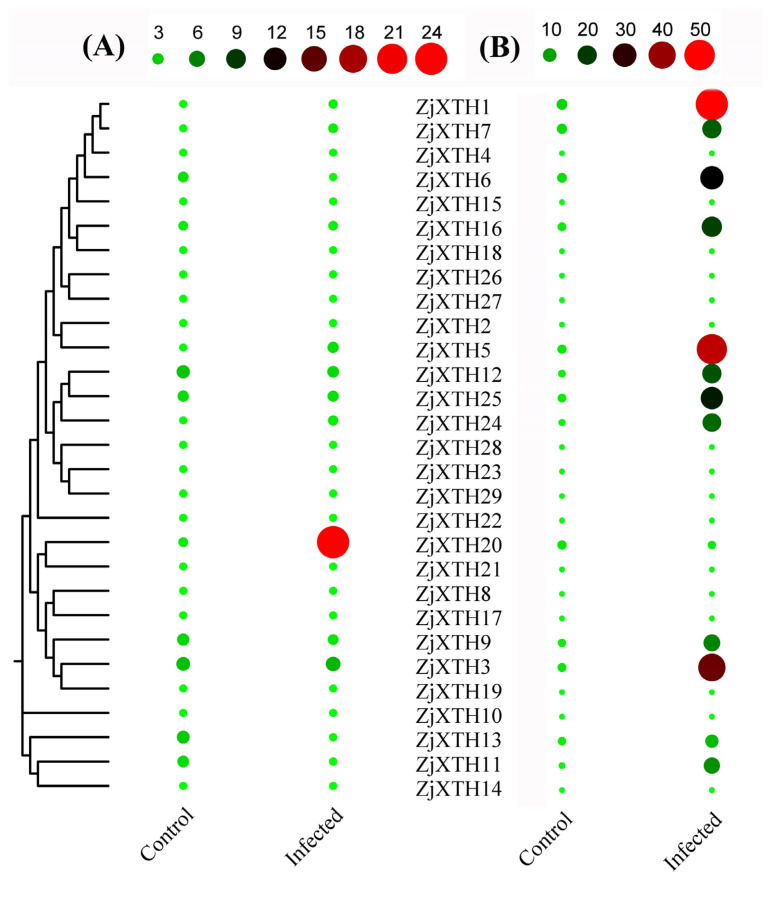
Heatmap of 29 differentially expressed genes in Chinese jujube under biotic stress caused by jujube witches’ broom phytoplasma (JWB). (**A**) *Z. jujuba* ‘Huping,’ a sensitive cultivar, and (**B**) *Z. mauritiana* ‘Cuiming,’ a tolerant cultivar, were grafted onto the diseased ‘Jinsixiaozao’ (*Z. jujuba*). Phenotypic observations were conducted 21 weeks after grafting. The heatmaps display the average FPKM values of the genes, where deeper red indicates higher expression levels and deeper green represents lower expression levels.

To investigate the gene expression patterns of *ZjXTHs* under different biotic stresses, we analyzed the transcriptome RNA-seq data of *ZjXTHs* in response to jujube witches’ broom phytoplasma (JWB). *Z. jujuba* ‘*Huping*’, a sensitive cultivar (Figure 10A), and *Z. mauritiana* ‘Cuiming’, a tolerant cultivar (Figure 10B), were used for comparison, and expression heatmaps were generated. *ZjXTH1*, *ZjXTH3*, and *ZjXTH5* were upregulated following JWB stress in the infection-tolerant jujube cultivar, demonstrating the most rapid and pronounced response. These genes exhibited the highest expression levels, indicating their pivotal roles in the jujube witches’ broom phytoplasma stress response. Conversely, *ZjXTH6*, *ZjXTH7*, *ZjXTH11*, *ZjXTH16*, and *ZjXTH25* were downregulated under the infection-tolerant conditions, showing relatively stable expression during phytoplasma stress treatments in the tolerant cultivar. Furthermore, *ZjXTH20* exhibited negatively regulated expression patterns.

## 3. Discussion

Xyloglucan endotransglucosylases/hydrolases (*XTH*) genes have been thoroughly examined in several plants due to their crucial functions in plant growth and stress response [31]. Chinese jujube (*Z. jujuba*) is a plant of significant commercial value, but its growth and development are affected by several abiotic and biotic conditions, including salt [24], drought [32], cold [27], and phytoplasma infection [18]. However, jujube has not been examined in relation to this gene family.

According to our comprehensive identification methodology, 29 *XTH* genes were identified in the Chinese jujube genome and designated *ZjXTH-1* to *ZjXTH-29* [33]. The number of identified *XTH* genes is significantly lower than that in other species, such as *Glycine max* (61), *Solanum lycopersicum* (37), *Triticum aestivum* (71), and *Nicotiana tabacum* (56) [15,34,35,36]. The functional properties of these genes are closely associated with their structural and physicochemical features. This analysis revealed considerable variation among the 29 *ZjXTH* protein members in terms of protein sequence length, molecular weight, isoelectric point (pI), and intron–exon distribution. In *Z. jujuba*, this variation indicates a significant amount of diversity among the *XTH* family members. Additionally, most *ZjXTH* genes were predicted to be in extracellular space, while a few were located in the Golgi apparatus, vacuole, mitochondrion, and nuclear region. This contrasts with previous reports for other XTH protein members in other plant species, where the majority of XTH proteins were found in the plasma membrane rather than the extracellular space or other locations [37]. Furthermore, phylogenetic analysis indicated that *ZjXTH* protein families were clustered into four groups, similar to those observed for XTH proteins from cucumber [31]. Interestingly, the *ZjXTH* proteins belonging to the same group demonstrated similar gene structures and conserved sequence expression, which is consistent with the previously documented literature, suggesting that XTH members within the same group may exhibit analogous functionalities. Moreover, most of the *ZjXTH* genes exhibit two main conserved domains (Glyco_hydro_16 and XET_C). This suggests a potential evolutionary divergence, highlighting the role of the XET_C domain in the evolutionary trajectory of XTH proteins in *Z. jujuba*.

The phylogenetic classification of XTH proteins from *Z. jujuba*, *O. sativa*, *M. domestica*, and *A. thaliana* indicates that *ZjXTH* genes can be classified into four distinct groups. Previous research has classified *XTH* gene families into specific categories across different plant species. In tobacco, eight familial groupings were identified, whereas three groups were observed in peanut [38], barley, and sweet potato. In contrast, poplar displayed four distinct categories. *ZjXTH* genes were observed to cluster more closely with XTH proteins from *G. max* than *O. sativa*, suggesting a closer evolutionary relationship between the XTH proteins in *Z. jujuba* and those of *A. thaliana* rather than *O. sativa*. A chromosomal localization study revealed that *ZjXTHs* were unevenly distributed across 9 of the 12 chromosomes of *Z. jujuba*. Previous studies have demonstrated that a group of gene functions shows significant conservation across several plant species. Therefore, it is essential to identify genuine orthologs across various plant species using synteny analysis. The results of the synteny study revealed a notable degree of synteny between the *Z. jujuba* genome and those of *G. max* and *A. thaliana*, showing 34 and 17 syntenic blocks of *ZjXTH* with *G. max* and *A. thaliana*, respectively. Conversely, only four syntenic blocks were discovered with *O. sativa* [39].

Protein–protein interaction analysis offers insights into potential functional associations among *ZjXTH* genes. *ZjXTH1* plays a positive role in the metabolism of polysaccharides, particularly glucuronarabinoxylan. Notably, *ZjXTH1* interacts with Expansin-B1 (EXPB1), a protein crucial for cell wall loosening, which facilitates processes that require cell wall modification. This interaction also contributes to the formation of root hairs, which are vital for efficient water and nutrient uptake. Meanwhile, the transcripts of some members exhibited strong responses to salt, low temperature, and drought stress. For example, the overexpression of *GsEXPB1* in soybean significantly increased the number of hairy roots, the root length, the root weight, and tolerance to salt stress [40]. Additionally, *ZjXTH3*, *ZjXTH9*, and *ZjXTH27* interact with Pectin Methylesterase 22 (PME22), which is crucial for the demethylesterification of pectin. The alteration of cell wall characteristics is essential to the plant’s response to diverse environmental conditions [41]. A further study indicates that the purified recombinant *CaPMEI1* protein not only inhibits PME, but also exhibits antifungal action against some harmful plant fungi. The virus-induced gene silencing of *CaPMEI1* in pepper increases vulnerability to *Xanthomonas campestris* pv., along with diminished expression of several defense-related genes [42]. Furthermore, transgenic Arabidopsis lines overexpressing CaPMEI1 show increased resistance to *Pseudomonas syringae* pv [43].

In several species, *XTHs* were predicted and identified as modulating light-associated genes and phytohormonal-regulated photomorphogenesis. Therefore, we predicted and analyzed the promoter cis-regulatory elements (CREs) in the 2k bp upstream of *ZjXTHs*. The results indicated that a total of 607 CREs were expected in all of the *ZjXTHs*. Growth and development regulatory elements, stress-responsive elements, and phytohormone-responsive elements comprised these CREs. In the promoter region of *ZjXTH* genes, the growth and development regulatory elements accounted for the highest proportion of CREs, with a total of 350 elements and 58% of the total CREs. CREs’ mild responsiveness was exceedingly prevalent in this category, comprising 308 elements and 88% of the category’s total elements. This was comparable to research conducted on Rosaceae sp. and cucumber [44]. Light significantly impacts a variety of plant processes, such as development, photosynthetic regulation, and circadian rhythm. *ZjXTHs* may promote chloroplast development, which may mitigate excessive light damage and enhance photosynthetic activity, as previously mentioned [45]. In turn, this leads to an increase in the conversion of light energy into chemical energy, which in turn leads to a greater accumulation of carbohydrates [46,47]. Nevertheless, this hypothesis can be further investigated by conducting additional experiments that involve the application of excessive light stress to Chinese jujube plants. Other elements in this category included the regulation of Zein metabolism, the expression of endosperm, the regulation of circadian control, and seed-specific elements.

The second category contains stress-responsive CREs, with 132 (22%) CREs predicted in the promoter regions of *ZjXTHs*. This category encompasses defense and stress responsiveness, anaerobic induction, low-temperature responsiveness, MYB drought-inducibility, and defense and stress responsiveness. The most prevalent elements in this category were anaerobic induction elements, which suggests that *ZjXTHs* play a critical role in the resistance of Chinese jujube plants to environmental stress [48]. While the third category of CREs identified in the promoter regions of *ZjXTHs* is associated with phytohormone response, there are a total of 125 (20%) predicted CREs. Methyl jasmonate (MeJA)-responsive elements are the most prevalent phytohormone-responsive elements, followed by abscisic acid-responsive elements, salicylic acid-responsive elements, gibberellin-responsive elements, and auxin-responsive elements. The identification of CREs in the promoter regions of *ZjXTHs* offers a unique perspective on the regulation of gene expression, with a particular emphasis on phytohormone signaling, stress response, and growth and development. This information can be employed to investigate gene expression and regulation, which will facilitate the enhancement of crop yields and the ability to withstand duress. The identification of molecular targets for the enhancement and propagation of Chinese jujubes can be facilitated by an understanding of the functions of these regulatory elements in *ZjXTH* genes.

Throughout growth and developmental phases, plants are subjected to numerous abiotic stresses, including low temperatures, salinity, and drought. The XTH enzyme is a crucial modulator of cell wall modification, playing a significant role in both the synthesis and degradation of cell wall components. It is essential for maintaining cell wall integrity and resilience, contributing to structural stability and stress tolerance under both optimal and adverse environmental conditions. Our research revealed that *ZjXTH1* and *ZjXTH5* may be essential for the regulation of salt tolerance in a jujube cultivar under lack of water and salt stress. The Arabidopsis gene *XTH23*, a homolog of *ZjXTH1*, also exhibited upregulation under salt stress, as evidenced by similar results [22]. Dhar et al. demonstrated that overexpressing *AtXTH22* enhanced cell division, elongation, and primary root growth [49]. Similarly, Takeda et al. reported that the application of xyloglucans to excised pea stems resulted in increased stem rigidity [50]. Numerous studies also underscore the critical role of the *XTH* gene family in bolstering plant stress tolerance. For instance, the overexpression of *CaXTH3* in tomato and *Arabidopsis thaliana* enhanced resistance to drought and salinity by modulating stomatal dynamics [19,51]. Conversely, Bi et al. found that *TaXTH17* negatively influenced plant resistance to salt and drought stress, suggesting a diverse functional spectrum within the *XTH* gene family [52]. Additionally, the heterologous expression of *XTH* in *Populus tomentosa* markedly increased total intracellular sugar content and yeast cell osmo-tolerance, indicating that *PtoXTH27* and *PtoXTH34* may be integral to osmotic stress response mechanisms [53]. Cold stress is a significant challenge for plants, with extreme instances potentially resulting in plant mortality. This research revealed that *ZjXTH1*, *ZjXTH5*, *ZjXTH18*, and *ZjXTH14* were expressed, with *ZjXTH1* demonstrating a significantly elevated expression level under freezing conditions. In cotton, the overexpression of the *GhXTH22* gene resulted in higher enzyme levels and activity, contributing to increased hemicellulose and cellulose accumulation. This accumulation enhances cell wall rigidity, which, in turn, influences cell wall biosynthesis and modulates the expression of associated genes [54]. While Tan et al. found that *MaXTH7* was identified in banana as a contributor to tolerance to low-temperature stress [55]. Collectively, these changes contribute to improved cold tolerance in plants. It is thus hypothesized that *XTH* positively regulates plant cold tolerance through modulations of cell wall rigidity. Phytoplasma-associated diseases have significantly jeopardized the cultivation of several commercially vital plants. This study demonstrated that the expression levels of *ZjXTH1*, *ZjXTH3*, and *ZjXTH5* increased in response to jujube witches’ broom (JWB) stress in the infected, tolerant jujube cultivar, exhibiting the most immediate and significant reaction. Li et al. found that *CsXTH04* is upregulated in response to citrus bacterial canker (CBC). Furthermore, the overexpression of *CsXTH04* increased susceptibility to CBC in transgenic citrus lines [30]. However, numerous questions remain unresolved and warrant further exploration, including the functional mechanisms and stress responses of *XTH* in the context of bacterial and fungal infections, as well as the pathways through which microbial presence induces *XTH* gene expression.

Taken together, this discovery underscores the dynamic nature of these genes and their potential involvement in the precise adjustment of Chinese jujube responses to the continuously evolving signals from their environment.

## 4. Materials and Methods

### 4.1. Genome-Wide Identification of XTH Genes

A query was searched on the NCBI database (https://www.ncbi.nlm.nih.gov/ accessed on 1 September 2024) to retrieve the protein sequences of the Chinese jujube (*Z. jujuba* ‘Dongzao’). The Hidden Markov Model (HMM) profiles for PF00722 (also known as Glyco hydro 16) and PF06955 (also known as XET C) were acquired from the Pfam database. These profiles correspond to protein domains associated with *XTH.* The profiles were used as queries in the HMMER3.0 program, using the default E-value, to perform a search throughout the database. The researchers used the PFAM and SMART databases to identify the conserved domains that are present in the putative Xyloglucan endotransglucosylase/hydrolase (*XTH*) proteins of *Ziziphus jujuba*. In order to provide a more comprehensive examination, only proteins including both the PF00722 and PF06955 domains were retained. Protein characterization was performed using the ExPASy database (https://web.expasy.org/protparam/ accessed on 5 September 2024) using the identified *ZjXTHs.* The protein sequences were analyzed to determine their predicted features, such as length, molecular weight (MW), instability index (II), isoelectric point (PI), aliphatic index (AI), and grand average of hydropathicity (GRAVY). The WoLF PSORT online database (https://wolfpsort.hgc.jp/ accessed on 7 September 2024) was employed for the purpose of predicting cellular localization [37].

### 4.2. Multiple Sequence Alignment and Phylogenetic Analysis

The XTH protein sequences of *A. thaliana* were obtained by accessing the Arabidopsis Information Resource (TAIR), whereas *OsXTHs* and *MdXTHs* were obtained via the Phytozome database (https://phytozome-next.jgi.doe.gov/ accessed on 10 September 2024). The phylogenetic tree was constructed using MEGA7.0 software, using the aligned sequences. The tree was constructed using the neighbor-joining (NJ) approach using the default parameters. A phylogenetic tree with 1000 duplicates was used to evaluate the reliability of the tree via the utilization of the bootstrap method. The graphical representation of the resulting tree is shown using ITOL v3 (https://itol.embl.de/ accessed on 10 September 2024).

### 4.3. Motif Analysis and Exon–Intron Structure of ZjXTHs

Utilizing the default settings, the MEME 20 suite (https://meme-suite.org/meme/ accessed on 10 September 2024) was used to ascertain the preserved motifs. TBTools software v2.102 was used to view the exons and introns of each *XTH* that were found using the GFF file of the *Z. jujuba* genome, which included gene structure information.

### 4.4. Chromosomal Locations, Synteny and Duplications Analyses of ZjXTHs

After obtaining the genomic data of *Z. jujuba* from NCBI, the location information of the *Z. jujuba XTH* genes was used to map each gene to its matching chromosomal position. Repetitive events of *ZjXTHs* family genes across several species were computed using MCscan. To compute the recurrent occurrences of *ZjXTHs* among *Z. jujuba* and the other three species (Arabidopsis, *G. max*, and *O. sativa*), the TBTools software v2.102 used a Dual Synteny Plotter.

### 4.5. Cis-Element Putative Promoter Regions and Ka/Ks Analysis

The functions and patterns of gene expression are influenced by the cis-elements present in the promoter regions. In order to ascertain the presence of cis-regulatory elements in the promoters of *XTH* genes in *Z. jujube*, NCBI-extracted 2 kb upstream sequences of the genes’ coding area were used as input in the PlantCARE program (https://bioinformatics.psb.ugent.be/webtools/plantcare/html/ accessed on 10 September 2024). TBtools software was then employed to screen and display the cis-elements. In order to approximate the evolutionary trajectory of the *ZjXTH* genes, the TBTools software v2.102 was used to compute the ratio of synonymous mutation rate to nonsynonymous mutation rate (Ka/Ks). The evolutionary calculation used *ZjXTH* protein findings above the 60 percent sequence similarity criterion that were obtained by blasting the proteins against the NCBI database. Following this, a tab-delimited text file containing the similarity data was generated in order to compute Ka/Ks using the TBTools software v2.102. The Ks value was used to approximate the divergence time in millions of years for each gene pair, with a substitution rate of 6.1 × 10^−9^ substitutions per site per year. To determine the divergence time (T), T was computed as T = Ks/(2 × 6.1 × 10^−9^) × 10^−6^ million years ago (Mya).

### 4.6. Protein Interaction Network and Gene Ontology

The protein interaction network was constructed using the STRING (https://cn.string-db.org/ accessed on 10 September 2024) database based on the orthologous genes between *Z. jujuba* and Arabidopsis. The predicted interaction network was visualized through Cytoscape software v3.10.3.

ShinyGO v0.77 (http://bioinformatics.sdstate.edu/go/ accessed on 11 September 2024) was used to obtain gene ontology (GO) annotation against *A. thaliana*. The *p*-value cut-off (FDR) at 0.01 is set to calculate GO enrichment.

### 4.7. Gene Expression Analysis of ZjXTH Genes Under Various Stresses

The RNA-seq expression data for the *Z. jujuba* were obtained from the National Center for Biotechnology Information database. The accession code for the data is GCF_000826755.1. The FPKM data of several tissues underwent hierarchical clustering analysis using TBTools software v2.102.

To measure the expression of *XTH* genes under stress tolerance, the RNA-seq data of our research previously reported were used. *Z. jujuba*. var. spinosa diploid and tetraploid, representing sensitive and tolerant types, respectively, were used in a salinity treatment, gradually applied at 50, 100, and finally 150 mM NaCL [24]. For drought treatment, PEG6000 was applied to diploid and tetraploid *Z. jujuba* var. spinosa seedlings, with concentrations of 5%, 10%, 15%, and finally 20% over 1-day intervals. For freezing treatment, branches were collected from the cold-sensitive cultivar ‘Dongzao’ and the cold-tolerant cultivar ‘Jinsixiaozao’. Some of the branches were placed at 4 °C for 10 h and used as controls, while others were treated and maintained at freezing temperatures of −10 °C, −20 °C, −30 °C, and −40 °C, also for 10 h. The xylem from these branches was then collected for RNA-seq library construction and sequencing [27]. For biotic treatment, ‘Cuimi’ (*Z. mauritiana* Lam. JWB tolerant) and ‘Huping’ (*Z. jujuba* JWB susceptible) scions were grafted onto the diseased ‘Jinsixiaozao’ (*Z. jujuba*), and the leaves were sampled for RNA-seq [18]. *XTH* expression data were analyzed using the FPKM values of assembled transcripts. FPKM values for different tissues were subjected to hierarchical clustering analysis using TBTools software v2.102. 

## 5. Conclusions

Our in-depth identification of the Xyloglucan Endotransglucosylase/Hydrolase (*XTH*) genes in Chinese jujube provides critical insights into their structure, function, and evolution. The *ZjXTH* genes are strategically positioned in the Group I/II category, which is crucial for cell wall dynamics. Phylogenetic analysis reveals similarities among plant species, while gene architecture emphasizes critical characteristics, such as the conservation of the Glyco_hydro_16 domain and Motifs 1 and 6, which regulate xyloglucan modification. For all *ZjXTHs*, our analysis of promoter cis-regulatory elements predicted 607 such elements. The interplay between Chinese jujube defense mechanisms and gene expression is exemplified by cis-acting elements, while environmental signal conversion is driven by TCA and TGACG motifs. *ZjXTH1* functions as a key regulator in the PPI network, enabling the precise regulation of *ZjXTH* gene expression in conjunction with EXPB1 and PME22. These results underscore the critical role of *XTH* genes in the growth, and adaptation of Chinese jujube, providing valuable insights for innovative applications in sustainable agriculture and beyond.

## Figures and Tables

**Table 1:** Identification and Characterization of *ZjXTH* genes in Chinese jujube.

						Gene Locations			Deduced Protein				
Gene Name	Protein ID	AA	bp	Chr	Signal Peptide (SP)	LOC	Start	End	MW(KDa)	PI	II	AI	GRAVY
*ZjXTH1*	XP_015899780.1	288	865	1	22	LOC107433023	2142963	2144333	32,104.7	5.24	38.45	60.31	−0.439
*ZjXTH2*	XP_015875377.2	297	899	1	34	LOC107412146	994499	995798	33,451.7	5.4	34.94	77.41	−0.292
*ZjXTH3*	XP_015868033.1	336	1014	12	25	LOC107405483	17889849	17892964	38,242.9	6.23	42.04	66.67	−0.475
*ZjXTH4*	XP_060672082.1	271	815	1	_	LOC107432095	2146026	2147442	30,453.67	5.26	36.72	55.79	−0.577
*ZjXTH5*	XP_015874851.4	296	877	1	30	LOC107411692	1002556	1004014	33,595.72	6.2	40.42	69.49	−0.401
*ZjXTH6*	XP_015899683.3	291	878	1	25	LOC107432937	2139826	2141413	32,396.09	5.42	38.32	64.71	−0.374
*ZjXTH7*	XP_015899503.3	292	867	1	25	LOC107432789	2137263	2138660	32,320.89	5.42	35.55	62.71	−0.398
*ZjXTH8*	XP_015898435.1	293	881	11	19	LOC107431919	7545976	7548758	33,835.31	9.38	43.23	62.25	−0.499
*ZjXTH9*	XP_048322840.2	310	928	12	27	LOC107428620	6457682	6460783	34,870.81	8.13	50.18	77.97	−0.137
*ZjXTH10*	XP_015892932.3	295	883	9	26	LOC107427100	15865101	15867060	34,701.11	8.56	37.48	66.1	−0.513
*ZjXTH11*	XP_015890447.3	293	882	7	30	LOC107425034	14371805	14373572	33,370.77	6.94	40.81	72.9	−0.31
*ZjXTH12*	XP_015887252.2	281	842	3	29	LOC107422327	13280316	13282451	32,220.62	9.17	36.22	74.95	−0.3
*ZjXTH13*	XP_015884034.2	304	911	1	33	LOC107419753	16657548	16659222	35,202.27	4.75	39.29	64.74	−0.518
*ZjXTH14*	XP_048328785.2	292	875	4	24	LOC107416878	25908816	25911364	33,164.31	5.41	32.69	69.42	−0.275
*ZjXTH15*	XP_015875279.3	292	882	1	27	LOC107412056	979197	980607	32,874.68	5.82	38.58	64.49	−0.418
*ZjXTH16*	XP_060671190.1	297	891	1	30	LOC107411876	969728	976681	3309.22	6.81	41.14	68.32	−0.367
*ZjXTH17*	XP_015874702.3	283	851	10	19	LOC107411602	17245584	17247133	31,912.58	6.79	48.08	64.13	−0.494
*ZjXTH18*	XP_048332201.1	294	884	1	29	LOC107406936	2149644	2151057	33,056.15	8.56	35.37	69.69	−0.296
*ZjXTH19*	XP_048320582.2	352	1045	6	35	LOC107403271	8140587	8143653	40,646.18	8.66	49.22	71.22	−0.422
*ZjXTH20*	XP_060668086.1	281	846	10	26	LOC132799666	6971363	6973149	32,513.47	5.48	35.04	73.49	−0.513
*ZjXTH21*	XP_060676394.1	279	837	9	_	LOC125424114	6979194	6981594	32,552.23	6.19	35.98	60.07	−0.713
*ZjXTH22*	XP_015898303.1	295	887	12	24	LOC107431811	25837449	25840155	34,115.81	8.62	36.67	67.76	−0.451
*ZjXTH23*	XP_015893429.2	300	903	4	32	LOC107427557	6965305	6966770	33,878.08	6.95	42.65	72.5	−0.288
*ZjXTH24*	XP_024928685.3	293	882	4	25	LOC107416347	6947614	6949140	33,775.19	9.47	36.74	68.26	−0.465
*ZjXTH25*	XP_048328992.1	311	936	1	_	LOC107412897	6046322	6048175	34,908	8.97	43.25	65.79	−0.406
*ZjXTH26*	XP_015875174.3	305	918	1	_	LOC107411968	1008911	1010755	34,241.17	4.98	43.09	67.51	−0.283
*ZjXTH27*	XP_060671181.1	288	867	1	26	LOC107411514	982063	988879	32,731.87	5.97	34.92	74.51	−0.29
*ZjXTH28*	XP_048336175.2	300	903	9	32	LOC125424038	23312053	23313630	34,157.58	8.54	39.98	73.13	−0.293
*ZjXTH29*	XP_048327183.1	300	903	4	32	LOC125421763	6952934	6954513	3392.92	8.54	42.4	71.5	−0.305

**Table 2:** Inter-specific gene duplication analysis of *ZjXTHs*.

Gene1	Gene2	Identity (%)	Ks	Ka	Ka/Ks	MAY
*ZjXTH1*	*ZjXTH7*	94.77	2.0593	1.6380	0.79	1.68
*ZjXTH2*	*ZjXTH5*	68.75	2.1000	1.8905	0.9	1.72
*ZjXTH4*	*ZjXTH6*	91.54	0.6033	0.0675	0.11	4.94
*ZjXTH17*	*ZjXTH8*	71.84	0.7556	0.8956	1.18	6.19
*ZjXTH23*	*ZjXTH28*	93.67	0.0545	0.0605	1.11	4.46
*ZjXTH12*	*ZjXTH29*	66.9	2.4965	1.2844	0.51	2.04
*ZjXTH15*	*ZjXTH26*	80.51	0.2084	0.3212	1.54	1.7
*ZjXTH16*	*ZjXTH18*	86.81	0.1081	0.1524	1.41	8.85

## Data Availability

Data are contained within the article.
